# Preconditioning, induced by sub-toxic dose of the neurotoxin L-BMAA, delays ALS progression in mice and prevents Na^+^/Ca^2+^ exchanger 3 downregulation

**DOI:** 10.1038/s41419-017-0227-9

**Published:** 2018-02-12

**Authors:** Serenella Anzilotti, Paola Brancaccio, Giuseppe Simeone, Valeria Valsecchi, Antonio Vinciguerra, Agnese Secondo, Tiziana Petrozziello, Natascia Guida, Rossana Sirabella, Ornella Cuomo, Pasquale Cepparulo, Andrè Herchuelz, Salvatore Amoroso, Gianfranco Di Renzo, Lucio Annunziato, Giuseppe Pignataro

**Affiliations:** 1 IRCCS SDN, Via Gianturco, 80131 Naples, Italy; 2 Division of Pharmacology, Department Neuroscience, School of Medicine, Federico II University of Naples, Via Pansini, 5, 80131 Naples, Italy; 3 Laboratoire de Pharmacodynamie et de Thérapeutique, Bâtiment GE, Faculté de Médecine, Université Libre de Bruxelles, route de Lennik 808, 1070 Bruxelles, Belgium; 4 Università Politecnica delle Marche, Ancona, Italy

## Abstract

Preconditioning (PC) is a phenomenon wherein a mild insult induces resistance to a later, severe injury. Although PC has been extensively studied in several neurological disorders, no studies have been performed in amyotrophic lateral sclerosis (ALS). Here we hypothesize that a sub-toxic acute exposure to the cycad neurotoxin beta-methylamino-L-alanine (L-BMAA) is able to delay ALS progression in SOD1 G93A mice and that NCX3, a membrane transporter able to handle the deregulation of ionic homeostasis occurring during ALS, takes part to this neuroprotective effect. Preconditioning effect was examined on disease onset and duration, motor functions, and motor neurons in terms of functional declines and severity of histological damage in male and female mice. Our findings demonstrate that a sub-toxic dose of L-BMAA works as preconditioning stimulus and is able to delay ALS onset and to prolong ALS mice survival. Interestingly, preconditioning prevented NCX3 downregulation in SOD1 G93A mice spinal cord, leading to an increased number of motor neurons associated to a reduced astrogliosis, and reduced the denervation of neuromuscular junctions observed in SOD1 G93A mice. These protective effects were mitigated in ncx3+/− mice. This study established for the first time an animal model of preconditioning in ALS and candidates NCX3 as a new therapeutic target.

## Introduction

Preconditioning (PC) is a phenomenon wherein a mild insult induces a cellular and tissue resistance to a later severe injury^1^.

Over the years numerous stimuli were described as possible PC inductors. Among these, hypoxic stimuli, bacterial toxins such as LPS, small seizures, volatile anesthetics, hyperthermia, and hypothermia^1^. Protection triggered by these stimuli is usually divided by a temporal point of view in acute, rapid PC, and long-lasting, delayed PC^1^. In particular, the delayed PC includes the involvement of a genomic reprogramming, which wavers, in most cases, in a downregulation or upregulation of proteins involved in the pathogenesis of the disease^2^. To date, although PC has been extensively studied in several neurological disorders such as Parkinson disease, brain ischemia, and epilepsy, no evidence has been provided on the existence of this PC neuroprotection strategy in amyotrophic lateral sclerosis (ALS).

The aim of this study was therefore to identify candidate stimuli and/or genes that can activate the pro-regenerative preconditioned state and to characterize the first PC model in ALS.

To this aim, we hypothesized that a sub-toxic dose of the cycad neurotoxin L-BMAA, an excitatory non-protein amino acid produced by cyanobacteria and associated with amyotrophic lateral sclerosis-Parkinson dementia complex (ALS-PDC), in Guam indigenous population, could be used as a PC stimulus. It is well known, in fact, that the toxin L-BMAA represents a possible cause of ALS linked to environmental factors. In fact, people who chronically feed foods rich in L-BMAA seem to contract the disease more frequently^3–5^. These data are supported by *in vitro* and *in vivo* experimental studies showing that this toxin may elicit ALS features^3–5^.

On this basis and assuming that a sub-toxic dose of the toxin could serve as a PC stimulus, experiments were conducted to verify: (a) whether the supposed PC stimulus could act as neuroprotectant, and (b) to elucidate the mechanisms underlying this phenomenon. Among the proposed putative mechanisms, it has been investigated the role of a plasma membrane ionic transporter, namely Na^+^/Ca^2+^ exchanger (NCX). Indeed, it is well known that alteration of calcium homeostasis is of significant importance in the pathogenesis of ALS^6,7^. In fact, in recent years it has been shown that the selective vulnerability of motor neurons in ALS may be due to the reduced capacity of these cells to buffer the excess of calcium ions that occurs in the course of the disease^7^. This concept arises from several studies carried out in humans and in cellular and experimental animal models of ALS in which Ca^2+^ binding proteins such as calbindin are reduced during the progression of the disease^8^. These findings are in good agreement with the demonstration that in patients and ALS mice, in those regions that precociously undergo to degeneration, such as facial, spinal, and hypoglossal motor neurons, the neuronal cytosolic Ca^2+^ buffering capacity is lower, whereas those areas that are more resistant to the disease display a greater cytosolic Ca^2+^ buffering capacity^9^. As NCX is one of the main mechanisms by which calcium and sodium ions can be extruded from the cell, we decided to investigate its role.

NCX is a membrane transporter that, by regulating the homeostasis of Na^+^ and Ca^2+^, participates to the evolution of several neurological disorders including brain ischemia, epilepsy, multiple sclerosis and Alzheimer disease^10–15^. To date three different isoforms, NCX1-3, and several splicing variants have been described within the CNS. The specific role of each isoform in ALS pathophysiology has not yet been determined, nevertheless some seminal works attribute to NCX3 a pivotal role in neuromuscular transmission impairment^16–18^. These information render this transporter particularly interesting as putative druggable target in ALS.

## RESULTS

### NCX3 transcript and protein expression decreased in spinal cord, brain stem, and gastrocnemius muscle of SOD1 G93A mice

To examine the role of NCX3 in ALS, we first determined NCX3 expression levels in affected CNS regions and in the gastrocnemius muscle of WT and G93A mice by real-time PCR and western blot analysis.

**Fig. 1:**
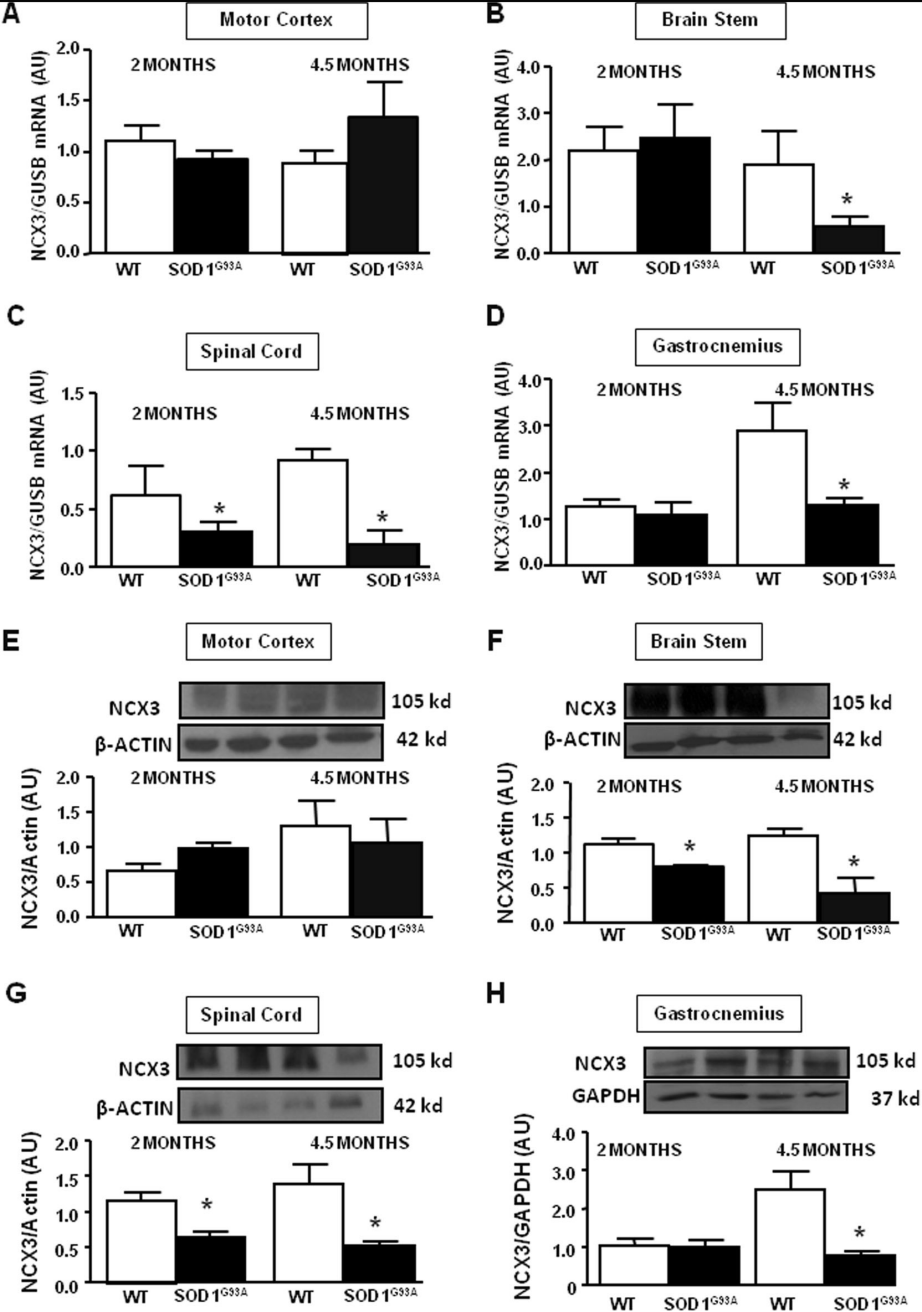
NCX3 mRNA and protein expression, arbitrary units (AU), in motor cortex, brain stem, spinal cord, and gastrocnemius of SOD1 G93A mice. NCX3 mRNA expression from motor cortex (**a**), brain stem (**b**), spinal cord (**c**), and gastrocnemius (**d**). The GUSB expression level was used for normalization. NCX3 protein expression, arbitrary units (AU), from motor cortex (**e**), brain stem (**f**), spinal cord (**g**), and gastrocnemius (**h**). β-actin expression level was used for E, F, G normalization, and GAPDH for H normalization. Data are expressed as mean ± SEM (*n* = 3–6 for each group of age). *P < 0.05 vs. respective wild type. Student’s *t*-test was used for the comparison between two mean groups

In particular, at 2 months of age, when SOD1 G93A mice were still asymptomatic, the expression of NCX3 mRNA appeared strongly reduced (50%) only in the spinal cord (Fig. 1c). Although, at 4.5 months, when SOD1 G93A mice were fully symptomatic, NCX3 mRNA expression was also reduced in brain stem area, spinal cord, and gastrocnemius (Fig. 1b-d). No changes in mRNA NCX3 levels were observed in motor cortex area in the asymptomatic and symptomatic phases (Fig. 1a).

The expression of NCX3 protein appeared significantly reduced in spinal cord, brain stem and gastrocnemius of SOD1 G93A mice in the fully symptomatic phase, 4.5 months (Fig. 1f–h) and in the brain stem and spinal cord of pre-symptomatic animals, 2 months. No changes were observed in motor cortex area (Fig. 1e).

### PC induced by sub-toxic treatment with the cycad toxin L-BMAA prevented NCX3 expression and activity downregulation in spinal cord and brain stem of SOD1 G93A mice

**Fig. 2:**
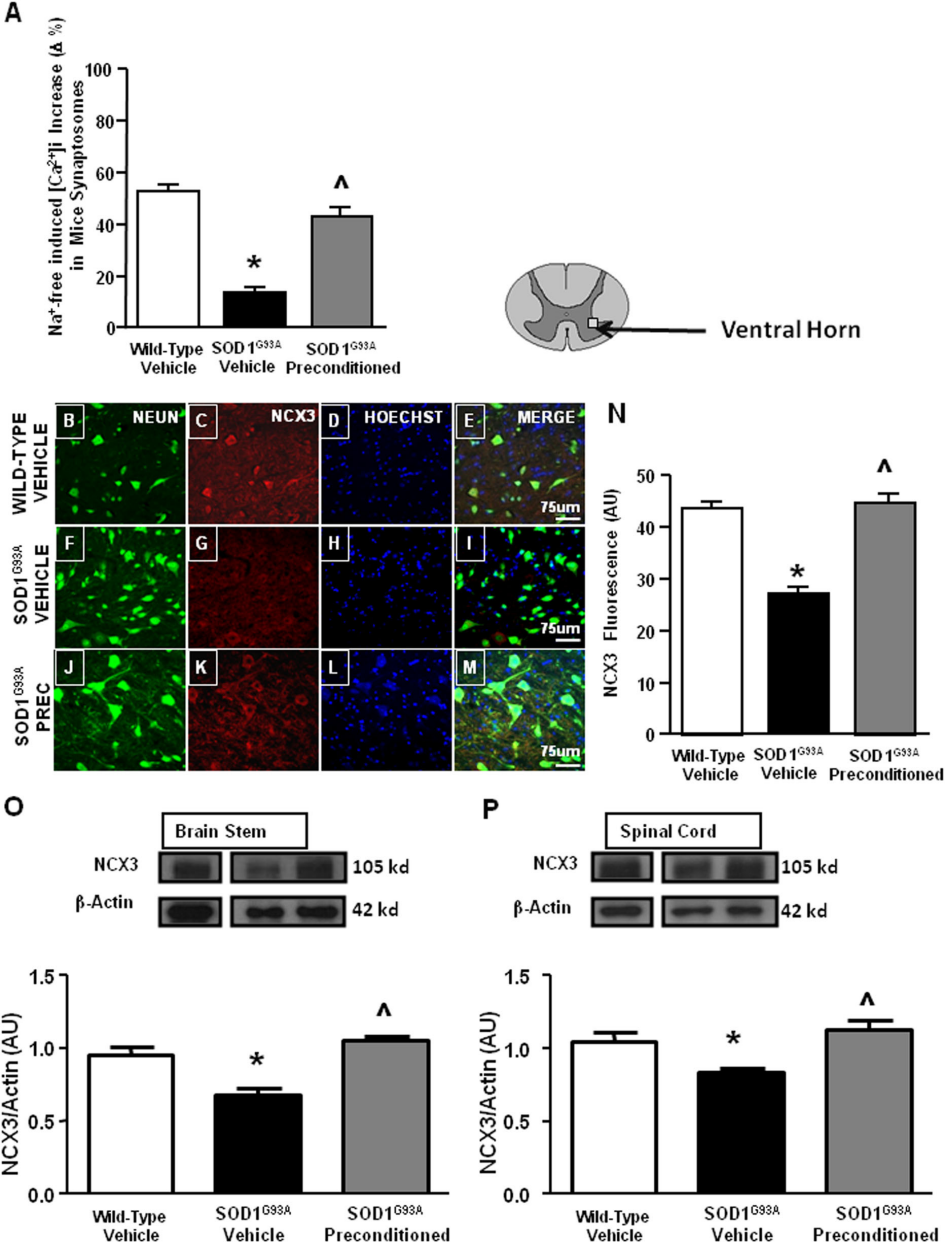
NCX3 immunolocalization, expression, and quantification of total NCX activity in SOD1 G93A mice subjected to L-BMAA-induced PC. Quantification of NCX activity as [Ca^2+^]i increase induced by Na^+^-free perfusion in Fura-2 AM-loaded spinal cord synaptosomes of adult wild-type mice treated with vehicle and symptomatic SOD1 G93A mice treated with vehicle or preconditioned with L-BMAA. *P < 0.05 vs. wild-type vehicle; ^^^*P* < 0.05 vs. SOD1 G93A mice (**a**). Double labeling of NCX3 and NeuN in spinal cord of pre-symptomatic wild-type mice (**b**–**e**), SOD1 G93A mice treated with vehicle (**f**–**i**) and SOD1 G93A mice treated with L-BMAA PC (**j**–**m**). Scale bar 75 μm. Quantification of NCX3 fluorescence as arbitrary units (AU). *P < 0.05 vs. wild-type vehicle; ^*P* < 0.05 vs. SOD1 G93A mice (**n**). Representative Western blotting and quantification of the effect of L-BMAA-induced PC on NCX3 protein expression, arbitrary units (AU), in brain stem (**o**) and spinal cord (**p**). *P < 0.05 vs. wild-type vehicle; ^*P* < 0.05 vs. SOD1 G93A mice. The β-actin expression level was used for normalization. Data are expressed as mean ± SEM (*n* = 5–6 for each group). *P < 0.05 vs. wild-type vehicle and SOD1 G93A preconditioned mice. *P* values were obtained using one-way ANOVA with Newman Keuls’s correction for multiple comparisons

To prevent NCX3 decrease observed in spinal cord and brain stem areas of SOD1 G93A mice, we settled up a PC protocol by an acute intracerebroventricular injection of L-BMAA toxin (4.5 mM/1 μl) and then, we evaluated NCX3 activity and expression by microfluorimetry, immunohistochemical, and western blotting analysis. SOD1 G93A mice treated with a sub-toxic dose of L-BMAA or saline, and a comparison group of wild-type mice treated with a sub-toxic dose of L-BMAA or vehicle, were killed 7 days after L-BMAA injection. To verify the effect of L-BMAA-induced PC on NCX3 activity, NCX reverse mode was evaluated by Na^+^-free-induced [Ca^2+^]_i_ increase in spinal cord synaptosomal preparations from L-BMAA-treated SOD1 G93A mice, vehicle-treated SOD1 G93A mice, and vehicle-treated SOD1 wild-type mice, all at 4.5 months. Na^+^-free-induced [Ca^2+^]_i_ increase was significantly reduced in synaptosomes from vehicle-treated SOD1 G93A mice compared to wild-type animals. Interestingly, L-BMAA PC significantly prevented the reduction of NCX activity registered in synaptosomes from SOD1 G93A mice (Fig. 2a).

Accordingly, further investigations by confocal microscopy experiments indicated that NCX3 expression increased in L-BMAA preconditioned SOD1 G93A compared to vehicle-treated SOD1 G93A mice (Fig. 2b–n). Interestingly, although NCX3 was present in all neuronal populations, its expression was prevalent in large polygonal-shaped neurons, such as motor neurons (Fig. 2b–m).

Finally, western blotting experiments confirmed that L-BMAA PC determined an upregulation of NCX3 expression in spinal cord and brain stem areas. In fact, in these two CNS regions, NCX3 protein expression was significantly higher in L-BMAA preconditioned SOD1 G93A mice than in vehicle-treated SOD1 G93A mice (Fig. 2o–p).

No effect on NCX activity and NCX3 expression were observed when wild-type animals were treated with sub-toxic dose of L-BMAA (Data not shown).

Notably, although NCX2 expression was reduced during ALS progression, PC was not able to induce any effect on NCX1 and NCX2 expression evaluated in brain stem and motor cortex of SOD1 G93A mice (Fig. S1).

### PC prevented motor neuron degeneration in ventral horn and nucleus facialis of SOD1 G93A mice

To further demonstrate that the protective effect of PC is correlated to NCX3, we generated SOD1 G93A/ncx3+/− mice by crossing SOD1 G93A mice with ncx3+/− mice.

**Fig. 3:**
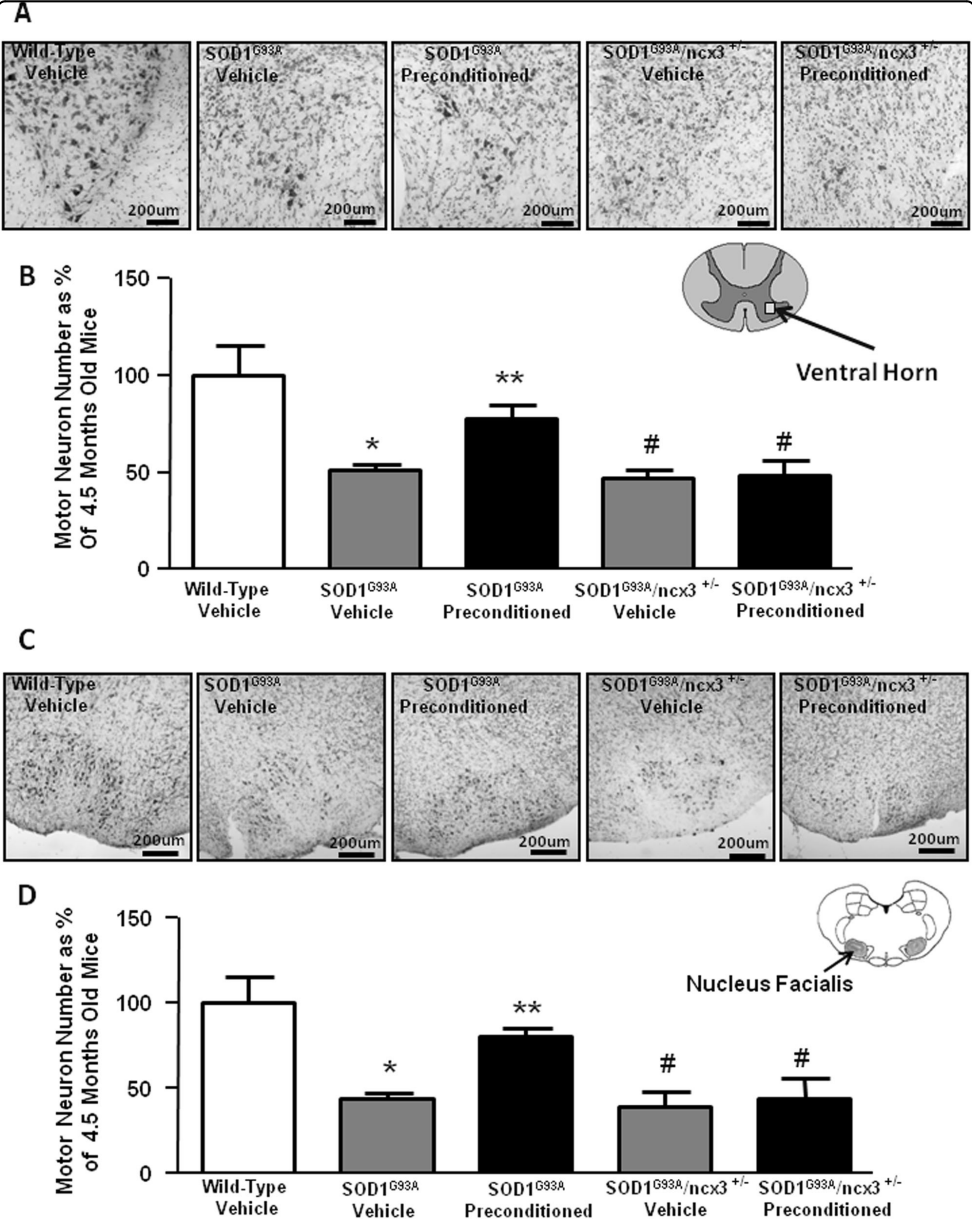
Effect of L-BMAA-induced PC on motor neurons survival. Representative image of Nissl stained in spinal cord (**a**). Scale bar 200 μm. Cell counting analysis of motor neurons expressed as the percentage of total motor neurons in spinal cord of 4.5 month-old SOD1 G93A mice treated with vehicle or l-BMAA compared to wild-type vehicle (**b**). Representative image of Nissl stained in nucleus facialis (**c**). Scale bar 200 μm. Cell counting analysis of motor neurons expressed as the percentage of total motor neurons in nucleus facialis of 4.5 month-old SOD1 G93A mice treated with vehicle or l-BMAA compared to wild-type vehicle (**d**). Data are expressed as mean ± SEM (*n* = 4 for each group). *P < 0.05 vs. wild-type vehicle **P < 0.05 vs. all other experimental groups. *P* values were obtained using one-way ANOVA with Newman Keuls’s correction for multiple comparisons

Sections of the spinal cord and brain stem nucleus facialis of these mice at the fully symptomatic phase of disease were stained with Nissl, and motor neurons were counted in the defined spinal cord and brain stem regions of interest. As ALS degeneration occurs preferentially in large motor neurons, the number of motor neurons with a perikaryal projection area of more than 200 μm^2^ was counted. PC by L-BMAA treatment preserved large motor neurons in the spinal cord (number of motorneuros per square mm in wild-type animals 292 ± 15; G93A mice 150 ± 3; G93A preconditioned mice 228 ± 2; G93A/ncx3+/− mice 136 ± 5; and G93A/ncx3+/− preconditioned mice 141 ± 7) and in the brain stem facialis nucleus (numer of motorneuros per square mm in wild-type animals 1800 ± 9; G93A mice 792 ± 3; G93A preconditioned mice 1442 ± 5; G93A/ncx3+/− mice 696 ± 9; and G93A/ncx3+/− preconditioned mice 793 ± 12) compared to vehicle-treated SOD1 G93A mice (Fig. 3a–d). Interestingly, PC failed to prevent the loss of motor neurons when applied to SOD1 G93A/ncx3+/− mice, thus suggesting that NCX3 contributes to the protection mediated by L-BMAA-induced PC (Fig. 3a–d).

### L-BMAA-induced PC reduced astroglia but not microglia proliferation in the spinal cord of SOD1 G93A mice

**Fig. 4:**
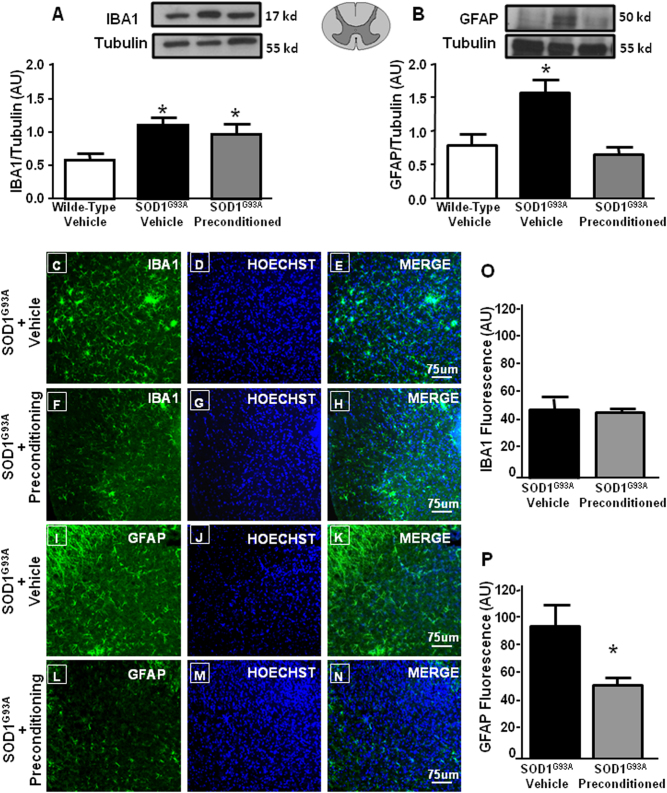
Effect of l-BMAA-induced PC on glial activation. Representative western blotting and quantification of the effect of l-BMAA-induced PC on IBA1 (**a**) and GFAP (**b**) expression, arbitrary units (AU). The tubulin expression level was used for normalization. Data are expressed as mean ± SEM (*n* = 4 for each group). *P < 0.05 vs wild-type vehicle. *P* values were obtained using one-way ANOVA with Newman Keuls’s correction for multiple comparisons. Single labeling of IBA1 in spinal cord of symptomatic SOD1 G93A mice treated with vehicle (**c**–**e**) and SOD1 G93A mice treated with l-BMAA PC (**f**–**h**). Single labeling of GFAP in spinal cord of symptomatic SOD1 G93A mice treated with vehicle (**i**–**k**) and SOD1 G93A mice treated with l-BMAA PC (**l**–**n**). Scale bar 75 μm. Quantification of IBA1 (**o**) and GFAP (**p**) fluorescence as arbitrary units (AU). *P < 0.05 vs. SOD1 G93A vehicle mice (Student’s *t*-test)

**Fig. 5:**
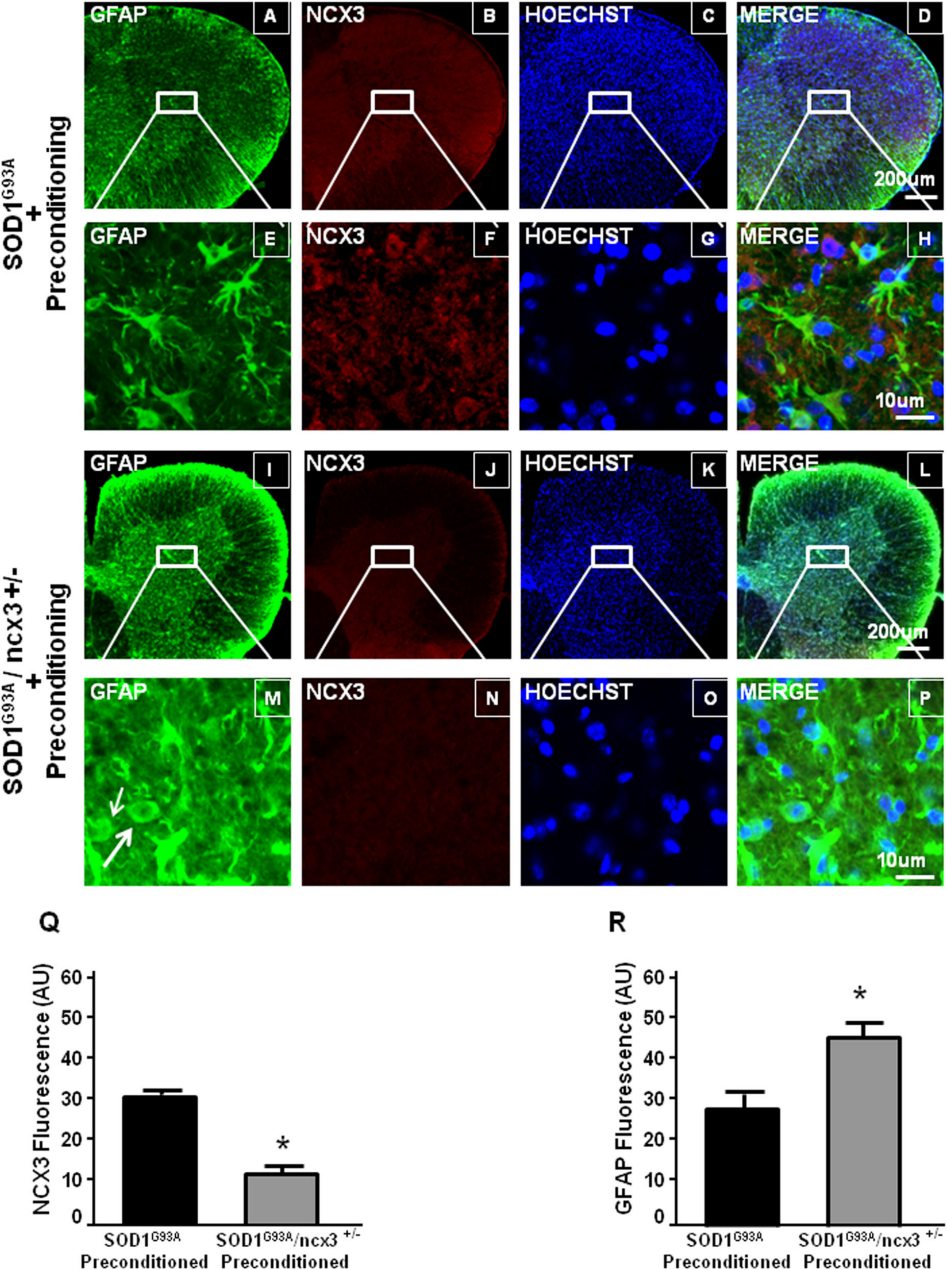
Contribution of NCX3 on astroglyosis reduction induced by l-BMAA-PC. Astroglyosis reduction induced by PC in SOD1 G93A mice (**a**–**d**, scale magnification 200 μm and at higher magnification **e**–**h**, scale 1 μm) is prevented in SOD1 G93A/ncx3+/− mice (**i**–**l**, scale magnification 200 μM, and at higher magnification **m**–**p**, scale 10 μm). Quantification of NCX3 (**q**) and GFAP (**r**) fluorescence as arbitrary units (AU). *P < 0.05 vs. SOD1 G93A preconditioned mice (Student’s *t*-test)

The motor neuron damage caused by expression of mutant human SOD1 induces the activation of astroglia and microglia, evaluated as GFAP and IBA-1 expression (Fig. 4). Interestingly, PC was able to prevent GFAP overexpression but not IBA-1 overexpression, thus suggesting that the reduced astrogliosis contributed to PC-induced protection. Indeed, GFAP-immunoreactive astrocytes were more abundant in vehicle-treated SOD1 G93A mice compared to preconditioned SOD1 G93A mice (Fig. 4i–p). Notably, when PC was induced in SOD1 G93A/ ncx3+/− mice, astrogliosis was no longer reduced (Fig. 5).

### L-BMAA-induced PC increased fully innervated end plates in neuromuscular junctions (NMJ) of SOD1 G93A mice

**Fig. 6:**
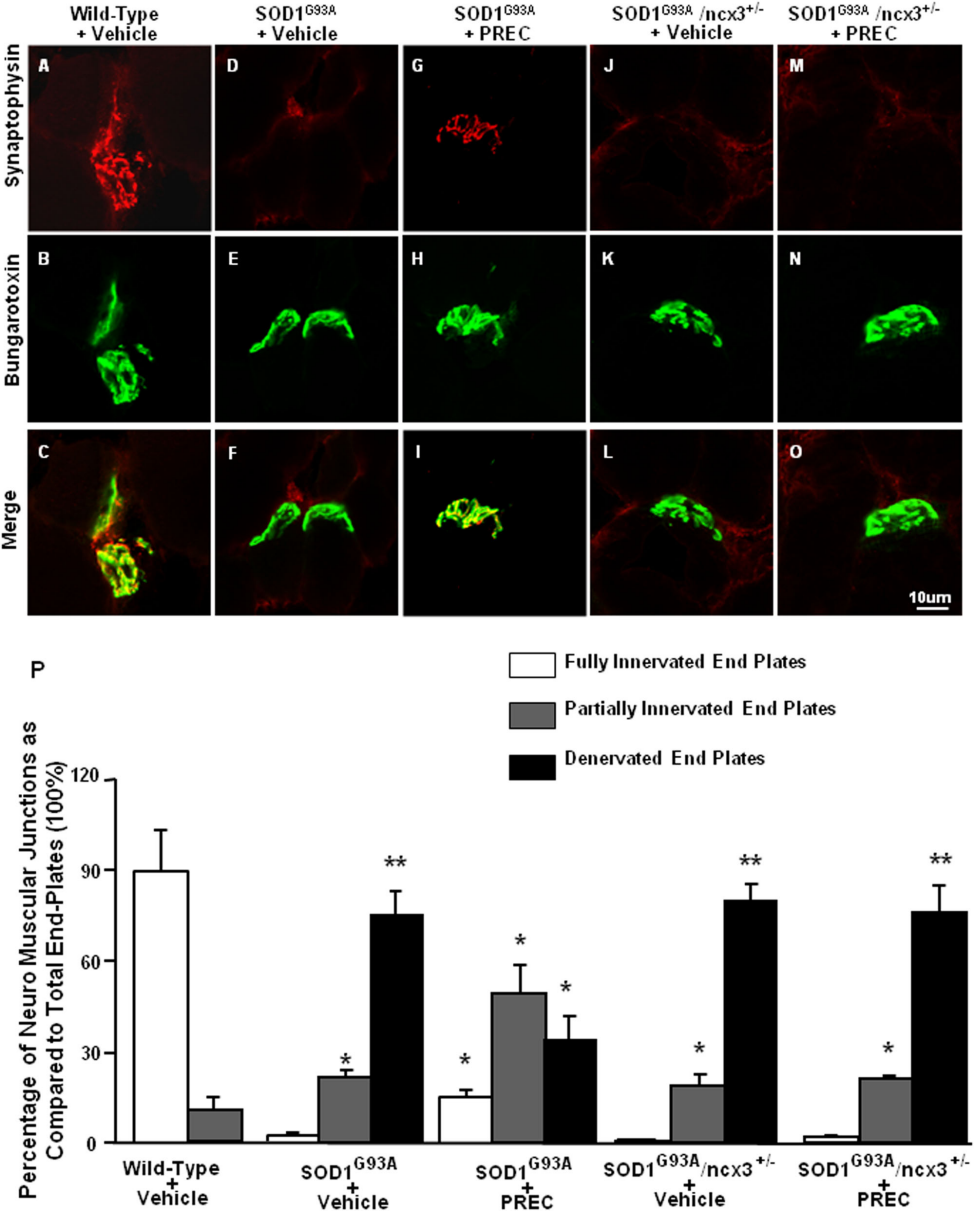
Effect of l-BMAA-induced PC on NMJs innervations of SOD1 G93A mice. Double labeling of synaptophysin and bungarotoxin of symptomatic wild-type mice treated with vehicle (**a**–**c**), SOD1 G93A mice treated with vehicle (**d**–**f**), SOD1 G93A mice treated with l-BMAA PC (**g**–**i**), SOD1 G93A/ncx3+/− mice treated with vehicle (**j**–**l**) and SOD1 G93A /ncx3+/− mice treated with l-BMAA PC (**m**–**o**). Scale bar 10 µm. Cell counting analysis of gastrocnemius NMJs of wild-type vehicle, SOD1 G93A vehicle, SOD1 G93A PC, SOD1 G93A/ncx3+/− vehicle and SOD1 G93A /ncx3+/− PC. The data are expressed as the percentage of 270 or more NMJs from each group. Data are expressed as mean ± SEM (*n* = 5 for each group). *P < 0.05 vs. respective wild-type vehicle. **P < 0.05 vs. all other experimental groups. *P* values were obtained using two-way ANOVA with Bonferroni’s correction for multiple comparisons

We first examined the effect of PC on NMJ innervation in 4.5 months old wild-type, G93A mice and G93A/ncx3+/− mice by assessing co-localization of fluorescently labeled pre-synaptic and post-synaptic NMJ markers in the gastrocnemius muscle (Fig. 6a–o). NMJs were classed as “fully innervated”, “partially innervated”, or “denervated”. As expected, by late-stage disease, a large proportion of NMJs exhibited complete (75.7 ± 7.0%) or partial (21.6 ± 2.4%) denervation in G93A mice, while only 2.5 ± 0.7% remained fully innervated. However, PC resulted in a significant increase in the proportion of fully innervated NMJs (15.4 ± 2.0%) and an equally marked decrease in the proportion of denervated NMJs (35.0 ± 7.0%) (Fig. 6p).

Notably, PC was not able to exert its protective effect in G93A animals crossed with ncx3+/− mice (% of denerveted end plates 76.3 ± 8.8) (Fig. 6p), thus indicating that the presence of NCX3 is necessary to mediate this protective effect.

### L-BMAA-induced PC improved survival rate and motor performances, delayed paralysis onset, and prevented body weight loss in SOD1 G93A mice during the development of the disease

**Fig. 7:**
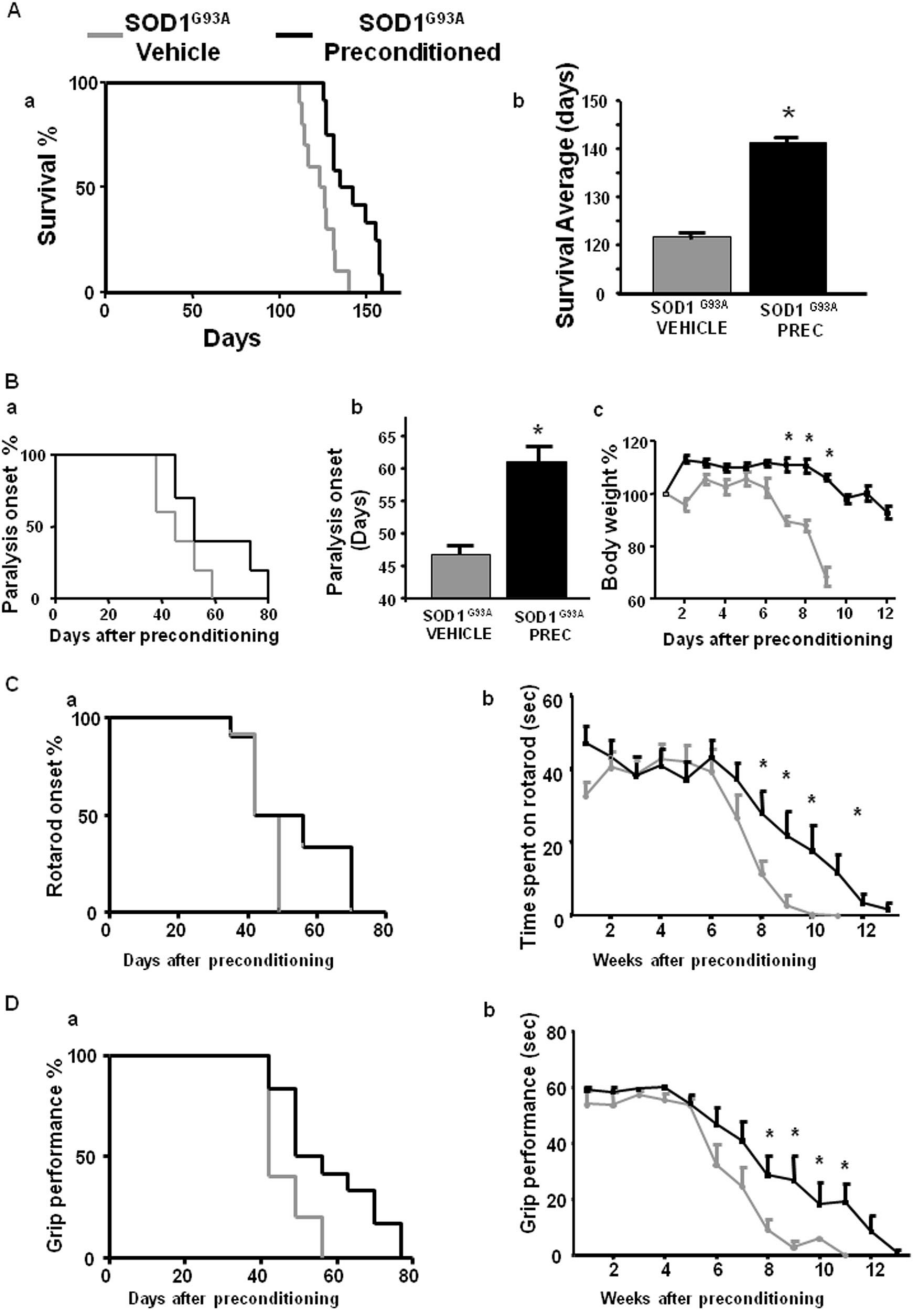
Effect of l-BMAA-induced PC on survival, paralysis onset, body weight reduction, and motor functions in SOD1 G93A mice. Survival curve of SOD1 G93A mice treated with vehicle compared to l-BMAA preconditioned SOD1 G93A mice (**A**a, b). Survival is expressed as percentage (**A**a) or in days (**A**b). Paralysis onset of SOD1 G93A mice treated with vehicle compared to l-BMAA preconditioned SOD1 G93A mice (**B**a, b). Paralysis onset is expressed in days after treatment with vehicle or l-BMAA PC (**B**a) or as percentage (**B**b). Percentage of body weight reduction in SOD1 G93A vehicle compared to SOD1 G93A mice preconditioned with l-BMAA (**B**c). Percentage of rotarod onset and time spent on rotarod by SOD1 G93A mice treated with vehicle compared to SOD1 G93A mice preconditioned with l-BMAA (**C**a, b). Grip performance of SOD1 G93A mice treated with vehicle compared to SOD1 G93A mice preconditioned with l-BMAA expressed as percentage (**D**a) or in seconds (**D**b). Data are expressed as mean ± SEM for **A**, b; **B**, b, c; **C**,b; and **D**, b. *n* = 10 for SOD1 G93A mice treated with vehicle, *n* = 12 for SOD1 G93A mice preconditioned with l-BMAA, *P < 0.05 vs. SOD1 G93A vehicle mice. Kaplan–Meier plot was used for **A**a, **B**a, **C**a, and **D**a. Student’s *t*-test was used for **A**b, **B**b, c, **C**b, **D**b

**Fig. 8:**
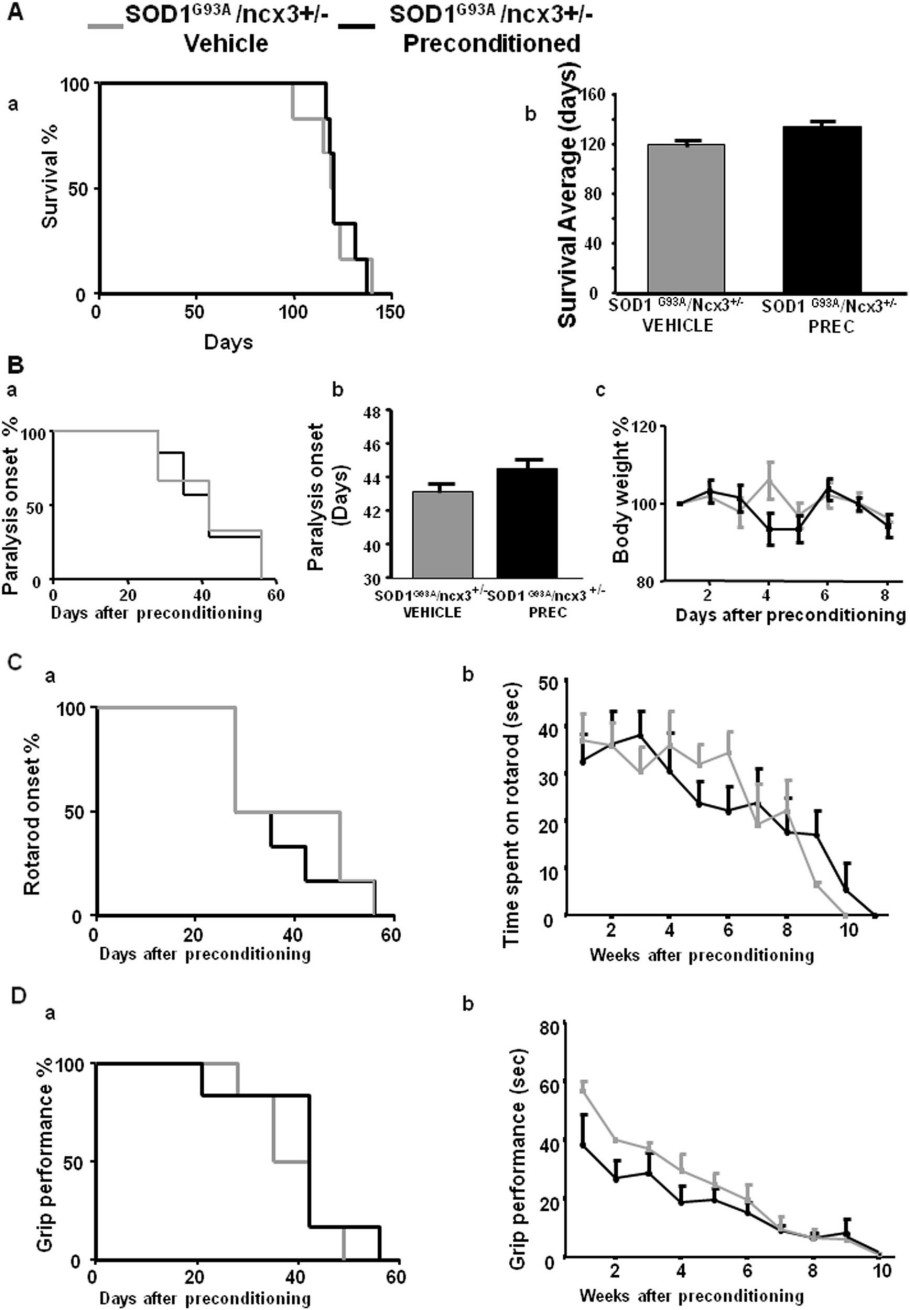
Effect of l-BMAA-induced PC on survival, paralysis onset, body weight reduction, and motor functions in SOD1 G93A /ncx3+/− mice. Survival curve of SOD1 G93A/ncx3+/− mice treated with vehicle compared to l-BMAA preconditioned SOD1 G93A mice (**A**a, b). Survival is expressed as percentage (**A**a) or in days (**A**b). Paralysis onset of SOD1 G93A mice treated with vehicle compared to l-BMAA preconditioned SOD1 G93A/ncx3+/− mice (**B**a, b). Paralysis onset is expressed in days after treatment with vehicle or l-BMAA PC (**B**a) or as percentage (**B**b). Percentage of body weight reduction in SOD1 G93A/ncx3+/− vehicle compared to SOD1 G93A/ncx3+/− mice preconditioned with l-BMAA (**B**c). Percentage of rotarod onset and time spent on rotarod by SOD1 G93A/ncx3+/− mice treated with vehicle compared to SOD1 G93A/ncx3+/− mice preconditioned with l-BMAA (**C**a, b). Grip performance of SOD1 G93A/ncx3+/− mice treated with vehicle compared to SOD1 G93A/ncx3+/− mice preconditioned with l-BMAA expressed as percentage (**D**a) or in seconds (**D**b). Data are expressed as mean ± SEM for **A**b; **B**b, c; **C**b; and **D**b. *n* = 6 for SOD1 G93A/ncx3+/− treated with vehicle or preconditioned with l-BMAA

L-BMAA PC prolonged the survival of SOD1 G93A mice compared to vehicle-treated animals (123.3 ± 3 vs. 142.5 ± 4 survival average days) (Fig. 7Aa, b). By contrast, L-BMAA PC was not able to prolong mice survival in SOD1 G93A/ ncx3+/− mice, (119 ± 6 vs. 123 ± 4 survival average days) thus showing that NCX3 contributes to PC effect (Fig. 8Aa, b).

The important role of NCX3 in mediating PC protection was confirmed by examining other parameters of ALS progression such as paralysis onset, body weight (Figs. 7b and 8b) rotarod (Figs. 7c and 8c) and grip performance (Figs. 7d and 8d). Indeed, the protective effect of PC was absent in SOD1 G93A/ ncx3+/− mice.

In fact, L-BMAA PC prevented the body weight loss observed in vehicle-treated SOD1 G93A mice from 7 weeks to 9 weeks after treatment (Fig. 7Bc). Conversely, in SOD1 G93A/ ncx3+/− mice, PC did not modify this parameter (Fig. 8Bc).

In a similar way, L-BMAA PC delayed the onset of disease by 14 days (46.4 ± 3 vs. 59.7 ± 5 days) in SOD1 G93A mice. By contrast, L-BMAA PC failed to delay paralysis onset in SOD1 G93A/ ncx3+/− mice (paralysis onset: 46 ± 3 days in SOD1 G93A mice; 60 ± 5 days in preconditioned SOD1 G93A mice; 44 ± 4 in SOD1 G93A/ ncx3+/− mice and 43 ± 5 days in preconditioned SOD1 G93A/ ncx3+/− mice) (Figs. 7Ba, b and 8Ba, b).

In addition, rotarod test showed that L-BMAA PC increased time spent on rotarod in SOD1 G93A mice but not in SOD1 G93A/ ncx3+/− mice (Figs. 7C and 8C).

Moreover, L-BMAA PC was able to attenuate the decline in grip performance in SOD1 G93A mice but not in SOD1 G93A/ ncx3+/− mice (Figs. 7D and 8D).

## DISCUSSION

The present study established for the first time an animal model for studying the possible protective role exerted by PC in SOD1 G93A mice based on the administration of a sub-toxic dose of the cycad neurotoxin L-BMAA. In addition, we demonstrated (a) that the cycad toxin L-BMAA, at this sub-toxic dose, improves survival rate and motor performances, delays paralysis onset and prevents body weight loss in SOD1 G93A mice during the development of the disease and that (b) the plasma membrane transporter Na^+^/Ca^2+^ exchanger 3, NCX3, contributes to the protection elicited by L-BMAA-PC thus representing a target for setting on new strategies in ALS intervention.

PC is a phenomenon wherein a mild insult induces a cellular and tissue resistance to a later severe injury^1,19^. Many different stimuli, named triggers, such as chemicals, ischemia, hypoxia, and hypothermia lead to PC. “Triggers” generate “transducers” and “effectors”, such as chemical mediators, neurotransmitters, and proteins, that elicit PC-protection^1^. In experimental models, protection through PC has been consistently demonstrated across multiple organ systems and in many different animal species, leaving no doubt on the existence of this phenomenon. For instance, in ischemic PC exposing an organ to brief ischemia induces temporary resistance to more severe ischemia, in the same or even a distant organ, i.e., remote conditioning^1,20–23^.

That a protein involved in calcium and sodium homeostasis maintenance could be linked to ALS pathophysiology was clearly conceivable from previous reports^6,24,25^. In fact, recent evidence suggest that abnormalities in cellular Ca^2+^ signaling are common features in the pathogenesis of a range of neurodegenerative disorders, including ALS^6,24,25^. It is well known that Ca^2+^ is one of the most relevant intracellular messengers, being essential in neuronal development, synaptic transmission and plasticity, as well as in the regulation of various transduction pathways in the brain^7^. In subtypes of ALS associated with the SOD1 mutation and in the sporadic disease, there have been several reports indicating the involvement of intracellular Ca^2+^ homeostasis disruption in ALS pathophysiology^6,7,24,25^. Combining conclusions from multiple animal models as well as cell culture models used to determine pathogenic mechanisms in ALS, the central insight is that selective vulnerability of MNs likely arises from a combination of several mechanisms; two of them, such as mitochondrial dysfunction and Ca^2+^ homeostasis are prominent^25^. By documenting the involvement and relevance of an alteration of Ca^2+^ homeostasis in ALS pathophysiology, it is possible to propose two scenarios: the first one is that motor neurons possess large number of transporters and ionic channels that, when activated, cause rapid Ca^2+^ influx, which, in part because of relatively weak cytosolic Ca^2+^ buffering, results in mitochondrial Ca^2+^ overload and strong ROS generation. In addition, it is possible to hypothesize that, in ALS affected tissues, the extrusion mechanisms, mainly represented by the high capacity-low affinity plasma membrane Na^+^/Ca^2+^ exchanger, are impaired and cannot provide an efficient removal of calcium ions. Results of the present study support this hypothesis. In fact, in line with these premises, we documented a reduction in NCX3 expression in motor neurons and muscle of asymptomatic G93A mice. Differently from NCX1 and NCX2, the other two CNS isoforms, the reduction in NCX3 expression was mitigated by PC treatment thus underlining the important role of NCX3 in ALS pathophysiology. In fact, in asymptomatic SOD1 G93A mice, PC treatment with L-BMAA did not modify the expression of NCX1 and NCX2 (data not shown). It should be underlined that NCX3 is not the only protein controlling intracellular Ca^2+^ concentration that is downregulated during ALS progression (see Supplemental Figure 1). In fact, we demonstrated that NCX3 is the only protein whose downregulation induced by ALS is prevented by PC. The effect on intra-synaptosomal Ca^2+^ reduction can, therefore, be ascribed, also to other proteins controlling intracellular Ca^2+^ concentration such as NCX2. The reduction of NCX3 protein expression was functionally mirrored by the reduction of the exchanger activity in synaptosomal preparations obtained from brain stem of SOD1 G93A mice. More importantly, in ncx3+/− mice the protection elicited by L-BMAA-induced PC was almost completely prevented, thus underlining the importance of this transporter in mediating PC-induced protection in ALS. Indeed, the increased NCX3 expression induced by PC is partially prevented in ncx3+/− mice, this is sufficient to prevent PC-induced protection. The fact that NCX3 may represent one of the effector of ALS PC is in line with previous works demonstrating that the genetic ablation of this transporter worsens the course of several neurological disorders such as brain ischemia, multiple sclerosis, Alzheimer disease and epilepsy^12,13^.

In conclusion, this study candidates NCX3 as a putative target in the strategy for alleviating ALS. The pharmacological activation or the overexpression of NCX3, by reducing the alteration in ionic homeostasis occurring in ALS, can mitigate motor neurons degeneration in ALS.

## MATERIALS AND METHODS

### Animal model

B6SJL-TgN SOD1/G93A(+)1Gur mice expressing high copy number of mutant human SOD1 with a Gly93Ala substitution [SOD1 G93A] and B6SJL-TgN (SOD1)2Gur mice expressing wild-type human SOD1 (WT)^26^ were obtained from Jackson Laboratories (Bar Harbor, ME, USA). Transgenic animals have been crossed with background-matched B6SJL wild-type female and selective breeding maintained each transgene in the hemizygous state. All transgenic mice were identified analyzing extracts from tail tips by staining for SOD1 as previously described^27^. To generate double-mutants carrying the NCX3+/− heterozygous mutation and the SOD1 G93A transgene (SOD1 G93A;ncx3+/−), SOD1 G93A male mice (mixed C57BL6-SJL background) were bred with ncx3+/− females (Sv129 background). NCX3 knockout mice (ncx3−/−) were generated by our research group as previously described^16^.

Overall, 120 male and female mice (50% each gender) housed under diurnal lighting conditions (12 h darkness/light) were used, 13 male and 12 female out of 120 animals were not included in the experimental groups as they died for unknown reasons. The number of female and male mice was balanced among all the experimental groups. Animals excluded were equally distributed among the experimental groups.

Experiments were performed according to the international guidelines for animal research and approved by the Animal Care Committee of “Federico II” University of Naples, Italy and Ministry of Health, Italy. All efforts were made to minimize animal suffering and to reduce the number of animals used.

### L-BMAA-induced PC procedure

L-BMAA was dissolved in saline solution (4.5 mM) and 1 μl intracerebroventricularly injected into the right lateral ventricle of 8-weeks-old mice on a stereotaxic frame using a stainless steel cannula connected to a Hamilton syringe through a PE10 tube (stereotaxic coordinates in mm with reference to the bregma were AP, −0.6; ML, −1.6; DV, −2.1)^28^. All mice were anesthetized with a gas mixture of 2% sevoflurane and 98% oxygen.

Usually, PC can be induced by toxic compounds used at 1/10 of their toxic concentration (i.e., LPS, hypoxia or tMCAO)^29,30^. Starting from this assumption, a concentration of L-BMAA ten times smaller than that used to mimic experimental ALS in vitro and in vivo, has been used.

### Tissue processing, immunostaining, and confocal immunofluorescence

Immunostaining and confocal immunofluorescence procedures were performed as previously described^31^. Animals were anesthetized and transcardially perfused with saline solution containing 0.01 ml heparin, followed by 4% paraformaldehyde in 0.1 mol/l PBS saline solution. Brains were rapidly removed on ice and postfixed overnight at +4°C and cryoprotected in 30% sucrose in 0.1 M phosphate buffer (PB) with sodium azide 0.02% for 24 h at 4°C. Next, brains were sectioned frozen on a sliding cryostat at 40 μm thickness, in rostrum-caudal direction. Afterwards, free floating serial sections were incubated with PB Triton X 0.3% and blocking solution (0.5% milk, 10% FBS, 1% BSA) for 1 h and 30 min. The sections were incubated overnight at +4 °C with the following primary antibodies: anti-NeuN (mouse monoclonal antibody; 1:500; Millipore, Milan, Italy), anti-NCX3 (1:3000; Swant, Bellinzona, Switzerland), anti-Glial Fibrillary Acidic protein (GFAP, rabbit polyclonal antibody; 1:500; Abcam, Cambridge, UK) and anti-ionized calcium binding adaptor molecule 1 (Iba1, rabbit polyclonal antibody; 1:500; Wako Diagnostic, Waco, VA, USA)

The sections were then incubated with the corresponding florescent-labeled secondary antibodies, Alexa 488/Alexa 594 conjugated antimouse/antirabbit IgGs (Molecular Probes, Invitrogen S.R.L., Milan, Italy). Nuclei were counterstained with Hoechst (Sigma-Aldrich, Milan, Italy). Images were observed using a Zeiss LSM700 META/laser scanning confocal microscope (Zeiss, Oberkochen, Germany). Single images were taken with an optical thickness of 0.7 m and a resolution of 1024 × 1024. In double-labeled sections, the pattern of immune reactivity for both antigens was identical to that seen in single-stained material. Control double-immunofluorescence staining entailed the replacement of the primary antisera with normal serum (data not shown). To minimize a possible cross-reactivity between IgGs in double immunolabeling experiments, the full complement of secondary antibodies was maintained but the primary antisera were replaced with normal serum or only one primary antibody was applied (data not shown). In addition, the secondary antibodies were highly preadsorbed to the IgGs of numerous species. Tissue labeling without primary antibodies was also tested to exclude autofluorescence. No specific staining was observed under these control conditions, thus confirming the specificity of the immunosignals.

Quantification of GFAP, NCX3, and Iba1 fluorescence intensity on tissue sections at the level of the spinal cord, was quantified in terms of pixel intensity value by using the NIH image software, as described previously^17,18^. Briefly, digital images were taken with ×40 or ×10 objective and identical laser power settings and exposure times were applied to all the photographs from each experimental set. Images from the same areas of each brain region were compared. Results were expressed in arbitrary units. Three sections from each mouse were analyzed, with *n* = 3 mice per treatment group.

To obtain an indirect measure of the amount of NCX3 in neurons, image analysis of NeuN was performed by NIH image software by measuring the intensity of fluorescent NCX3 immunolabeling in 50 NeuN positive neurons for each group. The intensity of NCX3 immunoreactivity was expressed in arbitrary units.

Standard Nissl staining was employed on coronal step serial sections from spinal cord and brain stem.

### Neuromuscolar junction (NMJ) analysis

Gastrocnemius muscle was removed and snap-frozen in liquid nitrogen-cooled isopentane. To study the NMJ, gastrocnemius muscle, sectioned in 10 μm thickness, in rostrum-caudal direction, was stained with a post-synaptic marker, α-Bungarotoxin, Alexa 488 conjugate (1:500), and a pre-synaptic marker, Synaptophysin (rabbit polyclonal antibody 1:500; Abcam, Cambridge, UK). NMJs were scored according to whether there was complete co-localization of pre- and post-synaptic markers (fully innervated), partial co-localization (intermediate innervation), or only post-synaptic labeling (fully denervated). All of the NMJ analyses were performed on 270 or more NMJs from each group^32^.

### Motor neurons counting analysis

Motor neurons were counted in the cervical spinal cord and in the brain stem facialis nucleus. Sections of each area were analyzed as previously described^33^. Frozen brain tissue and spinal cord were sectioned on a sliding cryostat at 20 μm, in rostrum-caudal direction. Four mice for each genotype and four slides from every mouse were analyzed.

Analyses were performed using image J software in Polygonal-shaped neurons larger than 20 μm with a well-defined cytoplasm, nucleus, and nucleolus^34^.

### Western blot analysis

Western blot analysis was performed as previously described^30^. Spinal cord, motor cortex and brain stem tissues were lysed in lysis buffer containing 50 mM Tris–HCl, pH 7.4, 150 mM NaCl, 1 mM EDTA, 1% Triton X-100, and protease and phosphatase inhibitors. Samples were subjected to SDS-polyacrylamide gel electrophoresis (SDS-PAGE) and immunoblotted with specific antibodies. Polyclonal antibodies were used against NCX3 (rabbit polyclonal; 1:2000; Swant, Bellinzona, Switzerland), anti- β-Actin (rabbit polyclonal 1:1000 dilution; Sigma-Aldrich, St. Louis, MO, USA). Immunoreactive bands were detected using ECL (GE Healthcare, Milan, Italy). The optical density of the bands (normalized for β-actin) was determined by Chemi-Doc Imaging System (Bio-Rad, Segrate, Italy)^35^.

### RT-PCR experiments

Tissues were quickly removed from mice, then immediately frozen on dry ice and stored at −80 °C until use. Total RNA was extracted with Trizol reagents, following supplier’s instructions (Life Technologies, Monza, Italy). The first-strand cDNA was synthesized with 2 µg of total RNA using the High Capacity cDNA Reverse Transcription Kit following supplier’s instruction (Life Technologies, Monza, Italy). Quantitative real-time PCR with TaqMan assays for NCX3 gene and glucuronidase beta (Gusb) as housekeeping were performed in a 7500 real-time PCR system (Life Technologies, Monza, Italy). Samples were amplified simultaneously in triplicate in 1 assay run. Changes in mRNA levels were determined as the difference in threshold cycle (ΔCt) between the target gene and the reference gene^36^.

### Purified synaptosomal preparation and [Ca^2+^]_i_ imaging

Spinal cord synaptosomes were purified on discontinuous Percoll gradients, as previously described^37^. Briefly, tissues were homogenized in a medium containing 0.32 M sucrose, 1 mM EDTA, and 0.25 mM dl-dithiothreitol (pH 7.4). Each homogenate was centrifuged at 1000×*g* for 10 min at 4 °C and the supernatant was diluted at 14 ml/g with sucrose medium (pH 7.4). Two ml of the suspension were placed onto 8 ml Percoll discontinuous gradient containing 0.32 M sucrose and 3%, 10%, 15%, and 23% Percoll (pH 7.4). After centrifugation at 32,000×*g* for 15 min at 4 °C, synaptosomes were recovered between the 15% and 23% Percoll bands, diluted five times with HEPES buffer medium containing (in mM): 125 NaCl, 2.5 KCl, 5 NaHCO_3_, 1.2 NaH_2_PO_4_, 1.2 MgSO_4_, 6 glucose, and 25 HEPES (pH 7.4), and centrifuged at 15,000×*g* for 15 min at 4 °C. Finally, the pellet was resuspended in 1 ml of medium B (145 mM NaCl, 3 mM KCl, 1.2 mM MgCl_2_, 10 mM glucose, and 10 mM HEPES, pH 7.4) and stored on ice. Protein content was determined by the Bradford method^38^. Percoll-purified synaptosomes were resuspended in medium B (1 mg/ml) and loaded with the ratiometric fluorescent Ca^2+^ indicator Fura-2 AM (6 µM)^12^ in the presence of 16 µM bovine serum albumin for 45 min at 37 °C. Dye-loaded synaptosomes were then washed by centrifugation, resuspended in medium B containing 1.2 mM CaCl_2_, and attached to poly-d-lysine-coated coverslips for 20 min at 37 °C. The coverslips were placed into a perfusion chamber (Medical System, Greenvale, NY, USA) mounted on the stage of an inverted Zeiss Axiovert 200 fluorescence microscope (Zeiss, Oberkochen, Germany) equipped with a ×40 oil objective lens. Experiments were carried out with a digital imaging system composed of MicroMax 512BFT cooled CCD camera (Princeton Instruments, Trenton, NJ, USA), LAMBDA 10-2 filter wheel (Sutter Instruments, Novato, CA, USA), and META-MORPH/METAFLUOR Imaging System software (Universal Imaging, West Chester, PA, USA). Synaptosomes were illuminated at 340 and 380 nm wavelength by a 100-W Xenon lamp (Osram, Berlin, Germany). The emitted light was passed through a 512 nm barrier filter. Images were digitized and analyzed using metafluor Imaging software. NCX activity was evaluated as Ca^2+^ uptake through the reverse mode by switching the normal Krebs medium to Na^+^-deficient NMDG^+^ medium named Na^+^-free, containing (in mM): 5.5 KCl, 147 NMDG, 1.2 MgCl_2_, 1.5 CaCl_2_, 10 glucose, and 10 Hepes-Trizma (pH 7.4).

### Evaluation of motor performance

Hindlimb grip test was conducted by placing the mouse on a grid (45 cm long × 28 cm large) upside-down (30 cm above a foam pad). The test was performed once a week and the latency to fall off the grid was also measured up to a maximum of 60 s.

Motor coordination and balance was assessed using a five-station mouse rotarod apparatus (Ugo Basile; Milan, Italy). In each station, the rod was 6 cm in length and 3 cm in diameter. Mice were trained to maintain balance at increasing speed up to a constant speed of 14 rpm for three consecutive trials. The test sessions were conducted by one rotarod trial administered once a week. In this session, the speed of rotation was increased from 4 to 14 rpm over 60 s. Mice had three trials on the rod, and the latencies to fall were measured once a week and then averaged. The maximum latency of 60 s was assigned to the mice that did not fall at all^39^.

Weekly evaluation of hindlimb paralysis was performed. Hindlimb paralysis was scored when the animal dragged one of its hindlimbs, and paralysis of a forelimb was scored when the mouse failed to use its forelimbs for walking or righting.

Body weight was measured immediately before each session of behavioral tests.

Disease endstage was defined by the inability of mice to right themselves within 20 s when placed on their sides.

### Statistical analysis

Data were evaluated as means ± SEM. Statistically significant differences among means were determined by one-way ANOVA followed by Student–Newman–Keuls post-hoc test for western blotting, cell counting, and real-time PCR analysis. Two-way ANOVA followed by Bonferroni post-hoc was used for motor performances test and body weight analysis. The Kaplan–Meier plot was used to evaluate survival, grip, rotarod and paralysis onset. Student’s *t*-test was used for two groups comparison. Statistical significance was accepted at the 95% confidence level (*P* < 0.05). Statistical analyses were performed by using GraphPad Prism 5.0 (La Jolla, CA, USA). All experiments were carried out in a blinded manner.

### Study approval

Experiments were performed according to the international guidelines for animal research and approved by the Animal Care Committee of “Federico II” University of Naples, Italy and Ministry of Health, Italy. All efforts were made to minimize animal suffering and to reduce the number of animals used.

## Electronic supplementary material

Figure S1

Figure S2
