# Supplementary figures and images for: Preconditioning, induced by sub-toxic dose of the neurotoxin L-BMAA, delays ALS progression in mice and prevents Na+/Ca2+ exchanger 3 downregulation
Source: Cell Death Dis. 2018 Feb 12;9(2):206. doi: 10.1038/s41419-017-0227-9 (PMC5833681; doi:10.1038/s41419-017-0227-9)

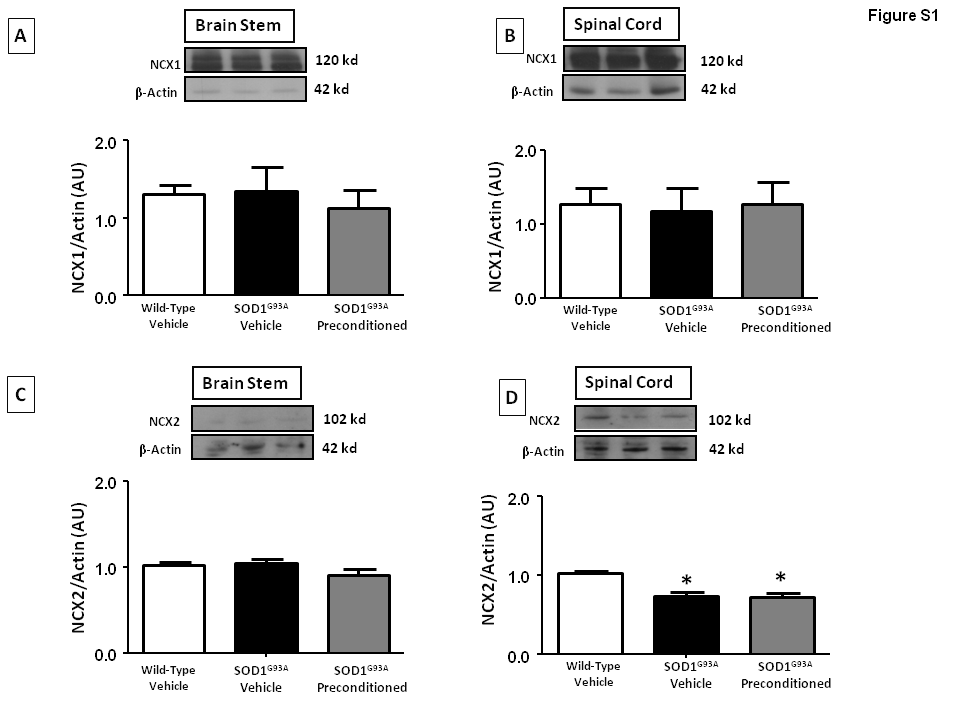

Supplement: Supplementary file 1 — Figure S1 [file 41419_2017_227_MOESM1_ESM.tif]
