# Supplementary material for: Preconditioning, induced by sub-toxic dose of the neurotoxin L-BMAA, delays ALS progression in mice and prevents Na+/Ca2+ exchanger 3 downregulation
Source: Cell Death Dis. 2018 Feb 12;9(2):206. doi: 10.1038/s41419-017-0227-9 (PMC5833681; doi:10.1038/s41419-017-0227-9)
Supplement: Supplementary file 2 — Figure S2 [file 41419_2017_227_MOESM2_ESM.docx]

**Supplementary Figure Legend**

**Figure S1:** NCX1 (A,B) and NCX2 (C, D) protein expression, arbitrary units (AU), from Brain Stem (A, C), and spinal cord (B, D). β-actin expression level was used for normalization. Data are expressed as mean ± SEM (n=3-6 for each group of age). *P<0.05 versus respective wild type. P values were obtained using 1-way ANOVA with Newman Keuls’s correction for multiple comparisons
